# Off‐the‐shelf femoral components consistently understuff the anterior compartment in kinematically aligned total knee arthroplasty: A prospective observational analysis from the FP‐UCBM knee study group

**DOI:** 10.1002/jeo2.70573

**Published:** 2026-04-16

**Authors:** Edoardo Franceschetti, Giancarlo Giurazza, Stefano Campi, Marco Edoardo Cardinale, Andrea Tanzilli, Pietro Gregori, Michele Paciotti, Matteo Pepe, Biagio Zampogna, Rocco Papalia

**Affiliations:** ^1^ Fondazione Policlinico Universitario Campus Bio‐Medico Roma Italy; ^2^ Department of Medicine and Surgery Research Unit of Orthopaedic and Trauma Surgery Roma Italy

**Keywords:** anterior femoral offset (AFO), kinematic alignment (KA), total knee arthroplasty (TKA), trochlear restoration

## Abstract

**Purpose:**

To assess the postoperative restoration of native anterior femoral offset (AFO) in kinematically aligned (KA) total knee arthroplasty (TKA) with the hypothesis that current off‐the‐shelf femoral components inadequately address the anterior compartment native anatomy.

**Methods:**

A prospective analysis was performed on 93 knees undergoing TKA with KA using GMK Sphere (Medacta) femoral components, excluding patients with posttraumatic osteoarthritis, radiographic signs of trochlear dysplasia ≥ type A of the Dejour classification, anterior femoral cortex notching or femoral component flexion >5° on postoperative lateral radiographs. After performing the anterior chamfer cut, the resected bone was measured with a caliper at the most prominent point of the trochlear groove, as well as the lateral and medial trochlear facets. After compensating for cartilage wear and saw blade thickness, the adjusted values were recorded as the native AFO. The thickness of the GMK Sphere femoral component at the trochlear surface corresponding to the middle of the anterior chamfer—specific to each implant size—defined the prosthetic AFO. AFO difference (AFOd) was defined as the difference between prosthetic and native AFO. *T*‐test and equivalence testing were performed to compare prosthetic and native AFO. Statistical significance was defined as *p* < 0.05.

**Results:**

AFO was consistently reduced across all regions, with a mean AFOd of −5.40 ± 2.40 mm at the medial trochlear facet, −2.32 ± 1.46 mm at the lateral facet, and −2.49 ± 1.11 mm at the trochlear sulcus (*p* < 0.001). The AFO was preserved in only 11.8% of cases at the medial trochlear facet, 29.0% at the lateral facet, and 18.3% at the sulcus. In none of the cases was the AFO increased.

**Conclusion:**

KA using off‐the‐shelf femoral components does not allow a full resurfacing of the trochlea, resulting in consistent offset reduction of the anterior compartment.

**Level of Evidence:**

Level IV, prospective observational study.

**Abbreviations**
AFO: anterior femoral offset
AFOd: AFO difference
ICC: intraclass correlation coefficient
KA: kinematic alignment
MA: mechanical alignment
SD: standard deviation
TKA: total knee arthroplasty

## INTRODUCTION

Kinematic alignment (KA) has emerged as an alternative to mechanical alignment (MA), accounting for individual anatomical variations [9, 12] and demonstrating improved clinical outcomes and patient satisfaction [22]. However, both techniques show similar rates of patellofemoral complications [7, 18, 23, 30]. This may be due to KA's current emphasis on restoring distal and posterior condylar anatomy [8], without specifically addressing the native anatomy of the trochlea. Specifically, in silico studies highlighted that current femoral components—originally designed for MA—frequently understuff the anterior compartment, irrespective of the alignment technique [27, 28]. A decreased anterior femoral offset (AFO) may cause patellar instability and quadriceps fatigue due to slackening of the patellar ligaments [25] and shortening of the lever arm of the extensor mechanism [6, 28], respectively.

Current efforts to restore the anterior compartment have largely focused on the proximal–anterior portion of the native and prosthetic trochlea [13], which is unlikely to represent the most critical segment responsible for patellofemoral joint mechanics, tracking and stability under dynamic conditions. Since patellar loads increase with knee flexion, in fact, the key trochlear segment is rather the area engaged by the patella between 30° and 70° of the femoral anterior arc [3, 15, 32]. Consistent with this, Campi et al. [1] recently defined the ‘mid‐flexion trochlear line’ (MTL) as the ‘functional’ anterior joint line, located 45° from the posterior condylar line and corresponding to the trochlear region engaged by the patella in mid‐flexion. To date, no intraoperative studies have examined this functionally relevant portion of the trochlea in the setting of a calipered KA technique [24, 29].

The primary aim of the current study was to evaluate whether KA can effectively restore the native AFO, with the hypothesis that current off‐the‐shelf femoral components are unable to accommodate the variability of the anterior compartment.

## MATERIALS AND METHODS

### Study design and participants

Institutional review board approval (IRB No. 32.19 OSS) was granted for this study and written consent was obtained from all participants. A total of 93 consecutive patients (102 knees) met the following inclusion criteria: end‐stage knee osteoarthritis (grade IV Kellgren–Lawrence) and undergoing TKA between September 2024 and March 2025 at authors’ Institution (Fondazione Policlinico Universitario Campus Bio‐Medico). The exclusion criteria were posttraumatic osteoarthritis, radiographic signs of trochlear dysplasia ≥ type A of the Dejour classification [5], anterior femoral cortex notching or femoral component flexion greater than 5° on postoperative lateral radiographs. No patients were excluded based on the severity of the patello‐femoral osteoarthritis. After applying these criteria, 86 patients (93 knees) were included in the final analysis.

#### Surgical technique and intraoperative measurements

**Figure 1:**
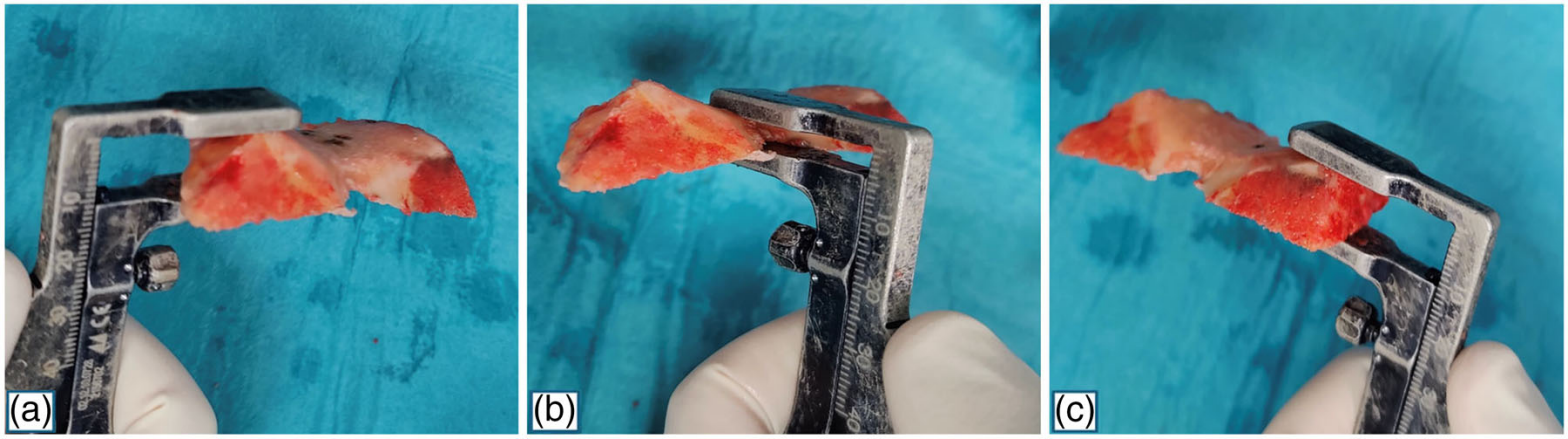
Intraoperative measurements of the lateral trochlear facet (a), trochlear groove (b) and medial trochlear facet (c).

The conventional calipered unrestricted KA technique was implemented in all the cases [14]. After osteophytes removal and following the distal femoral cut, a posterior‐referencing 4‐in‐1 cutting guide with 0° rotation was positioned in contact with the posterior femoral condyles. Adjustments were made to account for cartilage loss, ensuring that the femoral cuts were aligned parallel to the posterior condylar line, in accordance with the principles of calipered KA [14]. The appropriate anteroposterior size of the cutting block was verified, aiming to achieve an anterior cut flush with the anterior femoral cortex. After completing the posterior cuts—and confirming correct rotational alignment based on the thickness of the resected bone—the anterior chamfer cut was performed, and the resected bone was then measured with a caliper at the most prominent point of three locations, rounding to the nearest 0.5 mm: the lateral trochlear facet, medial trochlear facet and trochlear groove (Figure 1). Measurements focused on this region as it represents the functional part of the trochlea engaged by the patella during mid‐flexion—where the patella is anatomically constrained within the trochlea and functions as a key biomechanical lever [3, 15]. After compensating for cartilage wear (+2 mm) and saw blade thickness (+1 mm), the adjusted values were recorded as the native AFO. All surgical procedures were performed by the three senior surgeons and all measurements were taken independently by two authors (M.E.C. and G.G.) twice—once immediately after the cut and again at the end of the surgery.

**Figure 2:**
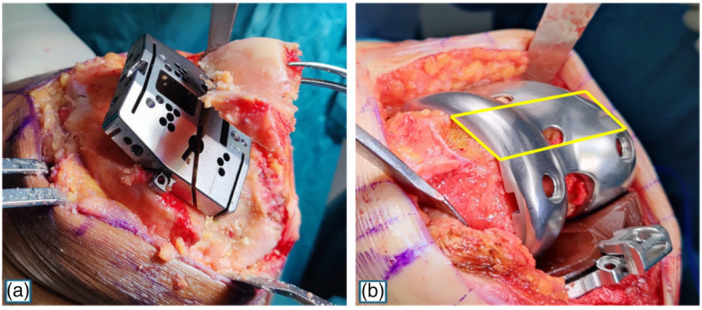
The anterior femoral offset difference was calculated by subtracting the bone thickness measured at the anterior chamfer cut (a) from the corresponding thickness of the prosthetic component at the same location (b).

The thickness of the GMK Sphere femoral component at the trochlear surface corresponding to the middle of the anterior chamfer—specific to each implant size (Table 1)—was provided by the manufacturer (Medacta, Switzerland) and defined the prosthetic AFO. To assess the restoration of AFO, the AFO difference (AFOd) was defined as the difference between the prosthetic and native AFO (Figure 2). An AFOd of 0 ± 1 mm was considered representative of a preserved AFO, while a positive and negative AFOd value indicated an increased and decreased AFO, respectively.

**Table 1:** Thickness of medial, lateral and sulcus of prosthesis (GMK Sphere—Medacta) divided by size.

Prosthetic size (GMK sphere medacta)	Trochlear sulcus thickness	Trochlear lateral facet thickness	Trochlear medial facet thickness
1/1+	3	8	7
2/2+	3	8	5
3/3+	3	9	4
4/4+	3	9	8
5/5+	3	9	6
6/6+	3	10	5
7	3	10	5

#### Data analysis

Descriptive statistics, including percentage, mean and standard deviation (SD), were calculated for all variables. Normality of data distribution was assessed using the Kolmogorov–Smirnov test. A *T*‐test was performed to compare prosthetic native and prosthetic AFO. Additionally, the two one‐sided Tests procedure was implemented to assess equivalence within a ±1 mm threshold. Measurement reliability was assessed using intraclass correlation coefficients (ICCs) for inter‐ and intraobserver agreement (0.91 and 0.93, respectively). Statistical significance was set at *p* < 0.05. Post hoc power analysis confirmed that the sample size (*n* = 93) provided over 99% power. All analyses were performed using STATA 18 Software (StataCorp LLC, Lakeway Drive).

## RESULTS

Demographic characteristics of the study population are reported in Table 2.

**Table 2:** Demographic characteristics of the 86 patients (93 prosthesis) included in the study.

Gender (*n*, %)	Male 39 (45.3%)
	Female 47 (54.7%)
Age (years ± SD)	69.9 ± 8.8
BMI (kg/m² ± SD)	28.4 kg/m² ± 5.7
Side (right or left knee)	54 Left
	39 Right
HKA (mean ± SD)	173.6° ± 4.5°
MPTA (mean ± SD)	86.2° ± 2.5°
LDFA (mean ± SD)	89.2° ± 2.8°

Abbreviations: BMI, body mass index; HKA, hip‐knee‐ankle angle; LDFA, lateral distal femoral angle; MPTA, medial proximal tibial angle.

**Figure 3:**
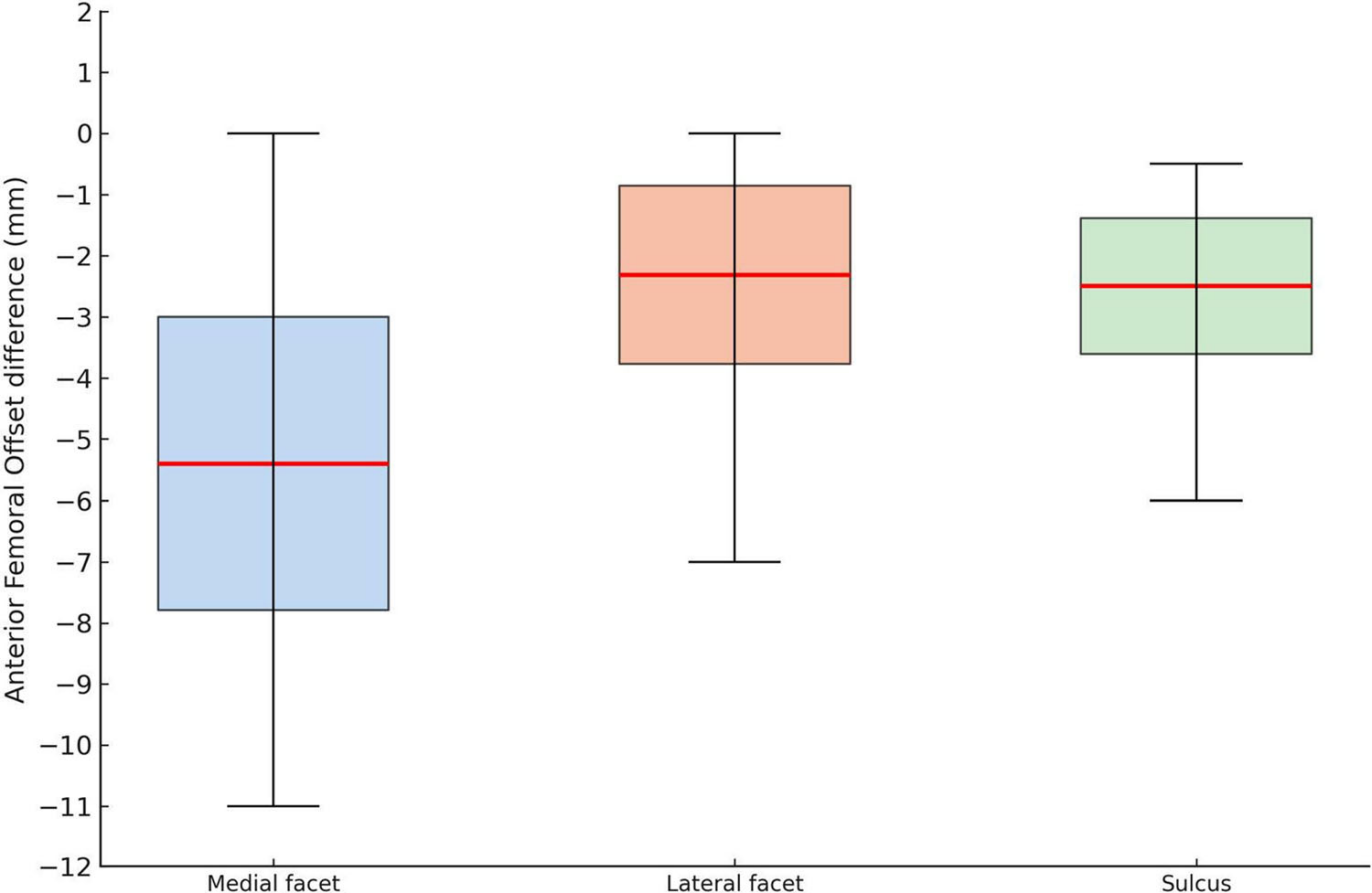
Boxplots illustrating the mean, standard deviation and total range of anterior femoral offset differences at the medial trochlear facet, lateral trochlear facet and trochlear sulcus.

Analysis of resection thickness showed a statistically significant reduction in AFO across all measured regions (*p* < 0.001): −5.40 ± 2.40 mm at the medial trochlear facet, −2.32 ± 1.46 mm at the lateral facet, and −2.49 ± 1.11 mm at the trochlear sulcus (Figure 3). Additionally, the two one‐sided test confirmed the absence of equivalence between prosthetic and native AFO within the predefined ±1 mm range (overall *p* = 0.650). The AFO was preserved in only 11.8% of cases at the medial trochlear facet, 29.0% at the lateral facet and 18.3% at the sulcus. In none of the cases the AFO was increased.

## DISCUSSION

The main finding of the current study was that the KA technique, paired with the conventional anterior femoral cut flush to the anterior cortex and with current off‐the‐shelf prosthetic components, fails to restore the AFO in the vast majority of patients.

Our findings, consistent with previous in silico studies [27, 28], showed a consistent understuffing, with a mean post‐op reduction of the AFO of −5.40 ± 2.40 mm at the medial trochlear facet, −2.32 ± 1.46 mm at the lateral facet, and −2.49 ± 1.11 mm at the trochlear sulcus. In all cases, femoral cuts were aligned parallel to the posterior condylar line, in accordance with the principles of calipered KA [14].

Importantly, these issues are not exclusive to KA. Even more pronounced understuffing is systematically observed with MA and with functional alignment (FA), as demonstrated by Rivière et al. [28] and Kafelov et al. [19], respectively. Collectively, these findings suggest that anterior compartment understuffing is primarily a consequence of prosthetic design, rather than the alignment technique employed.

The subsequent decrease in the quadriceps extensor moment of nearly 3% for every 1 mm of reduction in the lever arm [28] requires the quadriceps to exert greater force to extend the knee, potentially leading to complications such as quadriceps muscle overuse and fatigue [6], increased patellofemoral joint reaction force, accelerated wear and loosening of the patella component and painful patellar bone remodelling [27]. Additionally, a decreased AFO slackens patellar ligaments within a trochlea that already offers low constraint, thereby increasing the risk of patellar maltracking and instability [25]. Kafelov et al. [19] analysed the impact of restoring anterior compartment offset in FA at four flexion angles (0°, 30°, 70° and 90°). They found no significant effect of stuffing at 0° and 30°, and a negative impact on knee society score function and ROM—but not on kujala, forgotten joint score and patient satisfaction—of overstuffing >5 mm at 70° and 90°. Noteworthy, none of the cases in our series had any amount of overstuffing, making it highly unlikely that this ‘safe limit’ would ever be approached.

Notably, recent literature emphasising the importance of restoring the anterior compartment has primarily focused on the most anterior portion of the native and prosthetic trochlea [13]. However, the trochlear segment most critical to patellofemoral joint mechanics is the one engaged by the patella in mid‐flexion [3, 15, 32]. This more distal region—corresponding to approximately the 45° segment of the femoral anterior arch and referred to by Campi et al. [1] as the Mid‐flexion Trochlear Line—represents the true functional anterior joint line, which plays a critical role in patellofemoral kinematics and was specifically considered and evaluated intraoperatively in our study.

**Figure 4:**
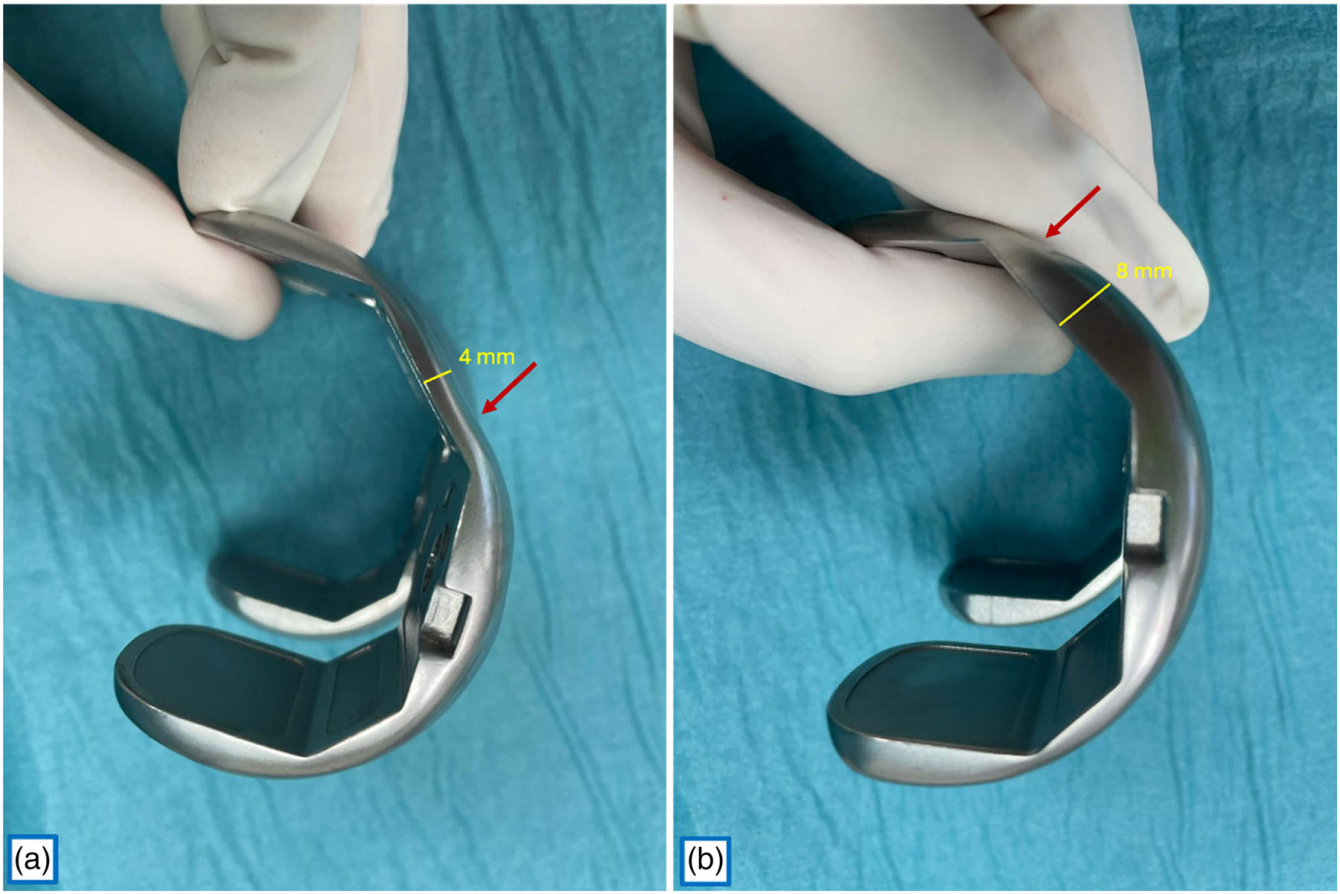
Prosthetic anterior femoral offset (AFO) at the level of the medial trochlear facet. Yellow line: thickness at the level of the midpoint of the medial trochlear facet; Red arrow: endpoint of the spherical curvature of the medial condyle. (a) size 3+: AFO 4 mm. (b): size 4: AFO 8 mm.

The notably larger SD of the AFOd on the medial side reflects the unique ball‐in‐socket design of the GMK Sphere femoral component. In some implant sizes, the midpoint of the medial trochlear facet precedes or aligns with the endpoint of the spherical curvature of the medial condyle; in others, the spherical surface ends more distally, leading to a thinner residual thickness at that level (Figure 4)**.** This geometric variability across sizes explains the greater variability in medial AFO reduction. Nonetheless, this region is likely not the most critical part of the trochlear surface, as the patella predominantly articulates with the trochlear sulcus and the lateral trochlear facet before 90° of knee flexion [3, 15].

Two main strategies may be employed to restore anterior compartment offset with standard implants—either before or after bone cuts. Before cutting, once the femoral component size is selected based on the anterior cortex, upsizing by one size may be considered if the femur's mediolateral dimensions allow it. This shifts the anterior chamfer cut 2 mm forward, and if an insufficient amount of bone is resected, the component can be easily downsized accordingly. In silico simulations [27] have shown that upsizing effectively restores AFO in the proximal 70% of the trochlea, though it may cause mild distal overstuffing. However, this is likely clinically negligible, as the patella articulates with the condyles—not the trochlear groove—during deep flexion [16, 17, 31], and functional outcomes are generally unaffected unless overstuffing exceeds the 5 mm safe limit [19].

Alternatively, in cases involving patellar resurfacing, the surgeon may calculate the AFOd after the standard anterior chamfer cut and then undercut the patella accordingly, relative to the thickness of the prosthetic component. This compensates for reduced femoral offset by increasing patellar offset. As with upsizing, increasing patellar offset across the flexion‐extension arc may risk paradoxical overstuffing in deep flexion. Nonetheless, its effect on the range of motion appears limited, with studies reporting a reduction of only 0.2°–1° per millimetre of anterior offset increase [19, 21, 26].

### Limitations

Our study presents limitations worth mentioning.

As a purely descriptive intraoperative study, it lacked both clinical and radiographic follow‐up. Future studies should integrate these aspects to reach a more comprehensive understanding of the patellofemoral biomechanics and their clinical implications.

Intraoperative measurements at the anterior chamfer cut were adjusted by 1 mm to account for saw blade thickness and by an additional 2 mm for cartilage wear, in line with the traditional calipered KA technique [14]. However, the actual saw blade thickness is typically slightly greater (approximately 1.34 mm), and studies by Giurazza et al. [10, 11] and Campi et al. [2] have shown significant variability in femoral cartilage thickness at the posterior and distal condyles—variability that likely also applies to the trochlear region. Despite that, considering a cartilage thickness ranging from 1.5 to 5 mm [2], these factors suggest that our reported amount of understuffing may in fact be underestimated, thereby reinforcing the validity of our findings.

Additionally, only knees implanted with GMK Sphere® (Medacta) femoral components using KA were analysed. Therefore, no direct comparison could be made with other alignment philosophies, such as mechanical or functional alignment. Nonetheless, similar findings have been reported in previous studies involving different implants and alignment techniques [4, 19, 20, 28].

## CONCLUSIONS

Off‐the‐shelf femoral prosthetic components consistently reduce the AFO. Tailored solutions should be adopted to accommodate interindividual variability in the anterior compartment, enabling KA to serve as a full resurfacing technique for both the tibiofemoral and—the so far neglected—patellofemoral compartments.

## AUTHOR CONTRIBUTIONS

Stefano Campi and Edoardo Franceschetti were responsible for data collection and conceptualisation. Giancarlo Giurazza had the idea for the study, was responsible for writing of the manuscript and qualified as corresponding author. Andrea Tanzilli and Matteo Pepe were responsible for data analysis. Pietro Gregori supervised data acquisition and analysis. Michele Paciotti and Marco Edoardo Cardinale were responsible for realisation of figures and tables. Biagio Zampogna and Rocco Papalia were responsible for reviewing and critically revise the manuscript. All authors have given final approval of the version to be published.

## CONFLICT OF INTEREST STATEMENT

The authors declare no conflicts of interest.

## ETHICS STATEMENT

The study was performed in accordance with the ethical standards as laid down in the 1964 Declaration of Helsinki and its later amendments. Institutional review board approval was obtained for this research (IRB n° 32.19 OSS). All patients provided legitimate informed consent.

## Data Availability

The data that support the findings of this study are available from the corresponding author upon reasonable request.
